# Supplementary material for: Mapping the prevalence of molecular markers of Plasmodium falciparum artemisinin partial resistance in Africa: a spatial-temporal modelling study
Source: medRxiv. 2025 Dec 23:2025.12.22.25342873. Preprint. [Version 1] doi: 10.64898/2025.12.22.25342873 (PMC12755267; doi:10.64898/2025.12.22.25342873)
Supplement: Supplement 2 [file media-2.pdf]

SUPPLEMENTAL FIGURES: MAPPING THE PREVALENCE OF MOLECULAR MARKERS OF *Plasmodium falciparum* ARTEMISININ PARTIAL RESISTANCE IN AFRICA: A SPATIAL-TEMPORAL MODELLING STUDY

Neeva Wernsman Young[1,2#], Cécile P. G. Meier-Scherling[1#], Gina Cuomo-Dannenburg[3,4], George A. Tollefson[1,2], Sean V. Connelly[5,6], Jacob Marglous[1,2], Isabela Gerdes Gyuricza[7], Kelly Carey-Ewend[5,6], Ronald Kyong-Shin[7,8], Zachary R. Popkin-Hall[6,9], Ayalew Jejaw Zeleke[10], Deus S. Ishengoma[11,12], Abebe A. Fola[2], Alfred Simkin[2], Karamoko Niaré[2], Jonathan B. Parr[6,7,13], Melissa Conrad[14], Lucy C. Okell[3], Shazia Ruybal-Pesántez[3,15], Oliver J. Watson[3], Jonathan J. Juliano[5,7,13,16†], Jeffrey A. Bailey[1,2*], Robert Verity[3*]

[1]Center for Computational Molecular Biology, Brown University, Providence, RI, USA
[2]Department of Pathology and Laboratory Medicine, Brown University, Providence, RI, USA
[3]MRC Centre for Global Infectious Disease Analysis, Imperial College, London, UK
[4]Department of Microbiology and Immunology, Rega Institute, KU Leuven, Leuven, Belgium
[5]MD-PhD Program, University of North Carolina, Chapel Hill, NC, USA
[6]Institute for Global Health and Infectious Diseases, University of North Carolina, Chapel Hill, NC, USA
[7]Curriculum in Genetics and Molecular Biology, University of North Carolina, Chapel Hill, NC, USA
[8]National Institute of Biomedical Research, Kinshasa, Democratic Republic of the Congo
[9]Department of Biology, Western Connecticut State University, Danbury, CT, USA
[10]Department of Medical Parasitology, School of Biomedical and Laboratory Sciences, University of Gondar, Gondar, Ethiopia
[11]Ifikara Health Institute, Ifikara, Tanzania
[12]National Institute for Medical Research, Dar es Salaam, Tanzania
[13]Division of Infectious Diseases, Department of Medicine, UNC School of Medicine, Chapel Hill, NC, USA
[14]Department of Molecular Microbiology and Immunology, Johns Hopkins School of Public Health, Baltimore, MD, USA
[15]Instituto de Microbiología, Universidad San Francisco de Quito, Quito, Ecuador
[16]Department of Epidemiology, Gillings School of Global Public Health, UNC, Chapel Hill, NC, USA

[#]These authors contributed equally. [*]Corresponding authors. [†]Senior author.

December 22, 2025

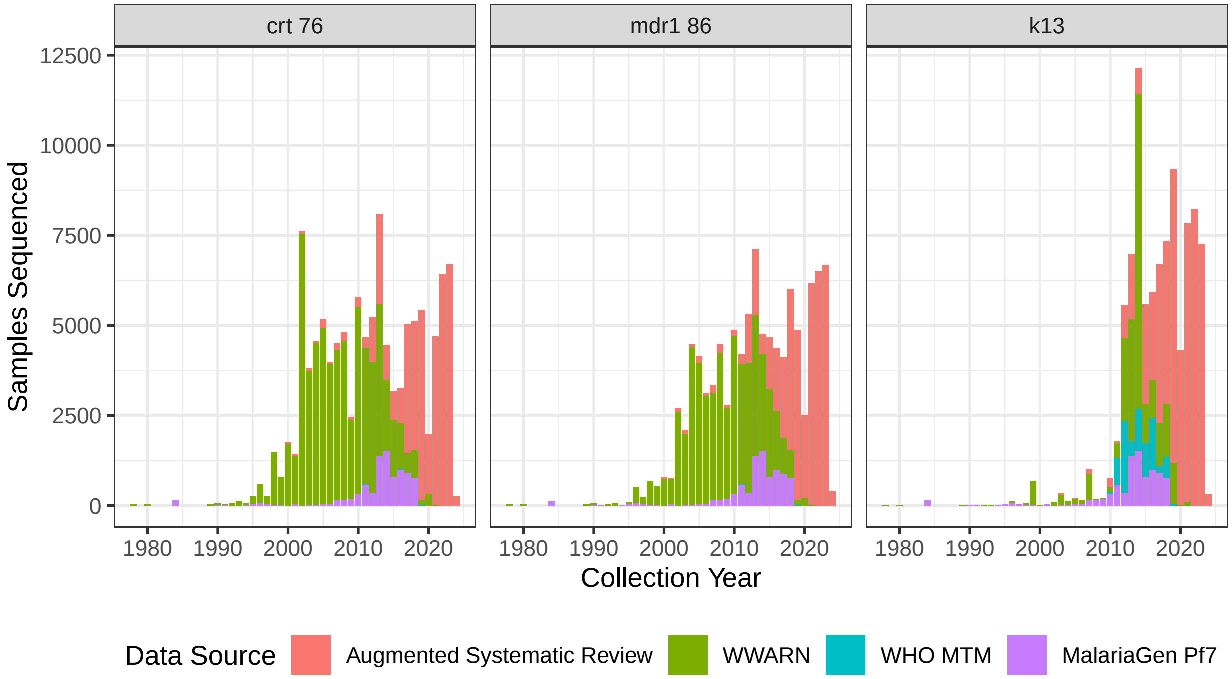

Figure S1: Number of samples in the combined dataset that were sequenced at each target position, from 1978 to 2024. For *k13* this includes all WHO validated and candidate ART-R mutation positions. Note that this is after ordered deduplication (Augmented Systematic Review ← WWARN ← WHO MTM ← MalariaGen Pf7).

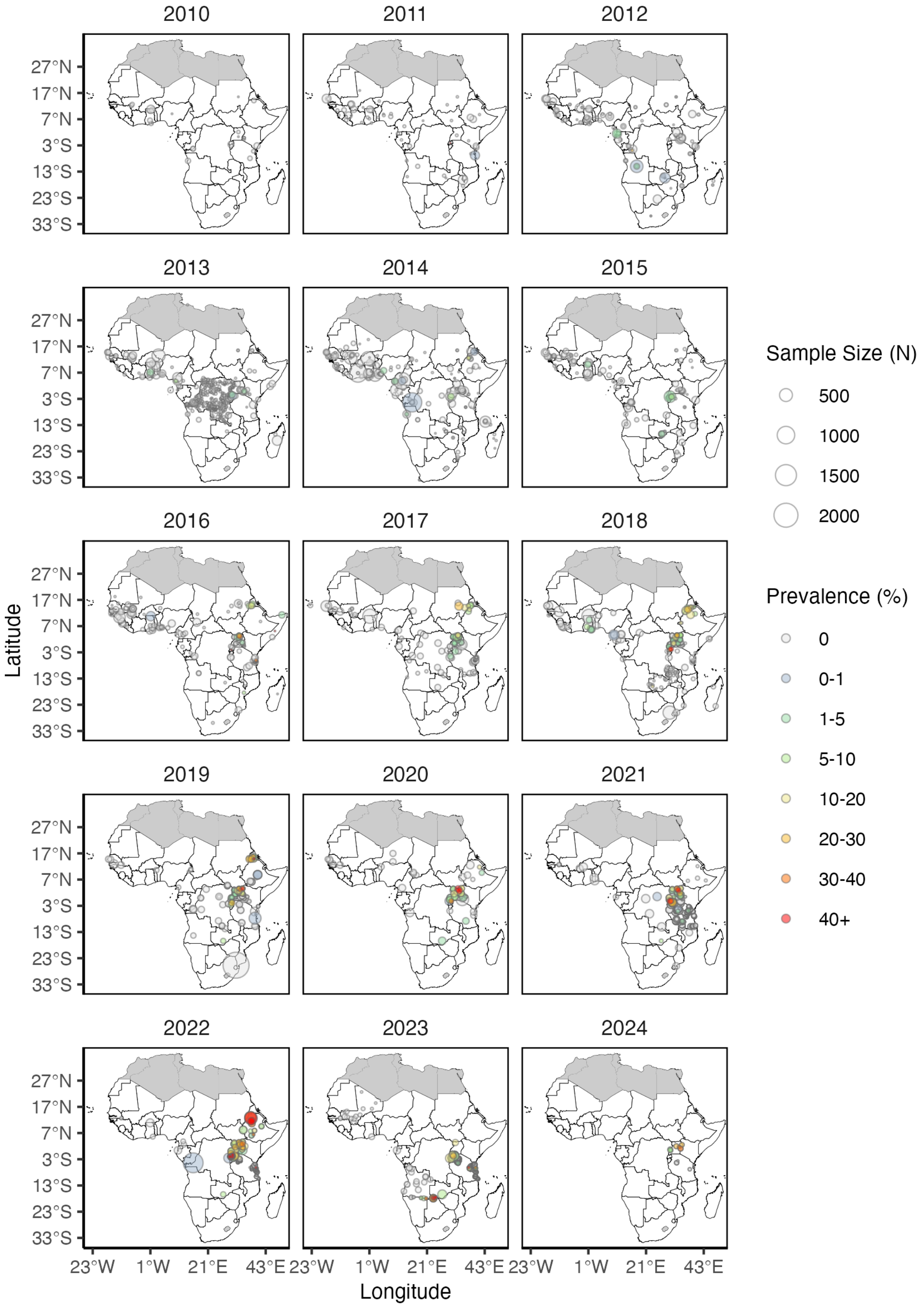

Figure S2: Observed prevalence of all WHO validated and candidate *k13* ART-R mutations combined across Africa from 2010 to 2024.

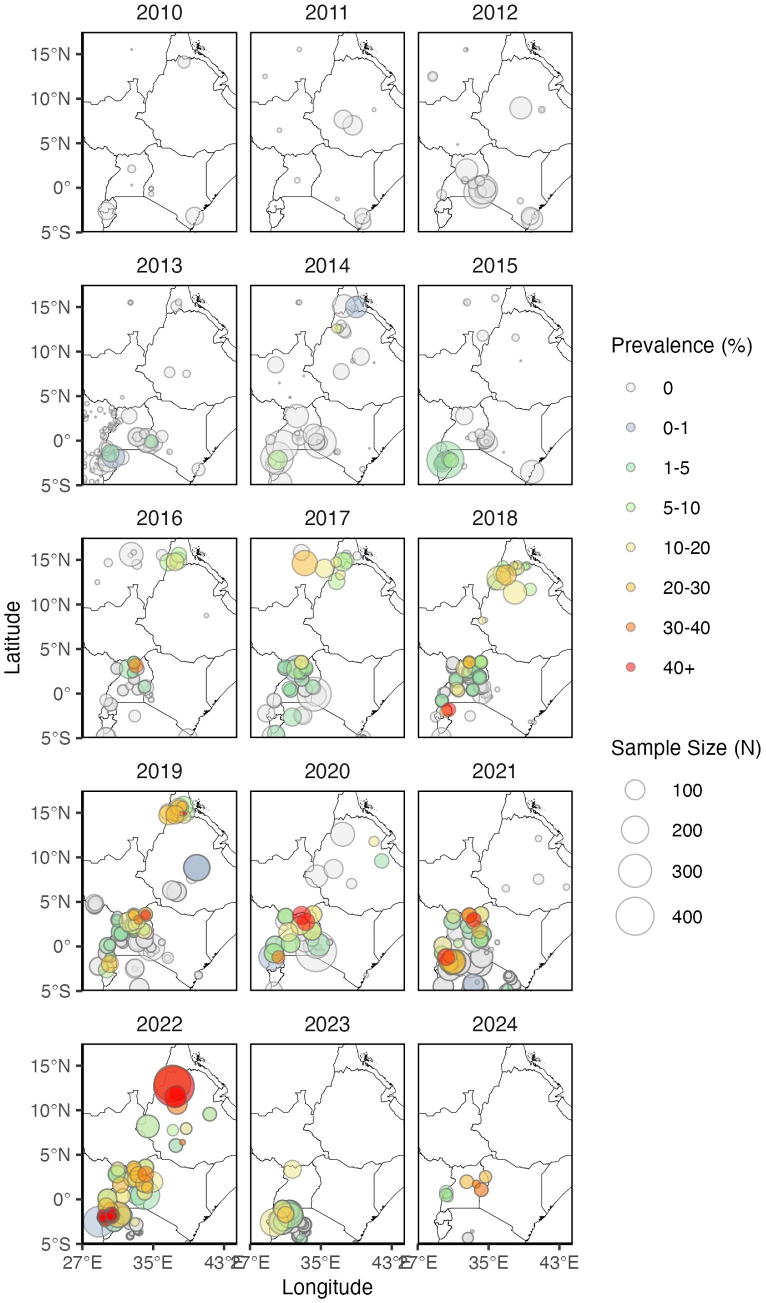

Figure S3: Observed prevalence of all WHO validated and candidate *k13* ART-R mutations combined across East Africa from 2010 to 2024.

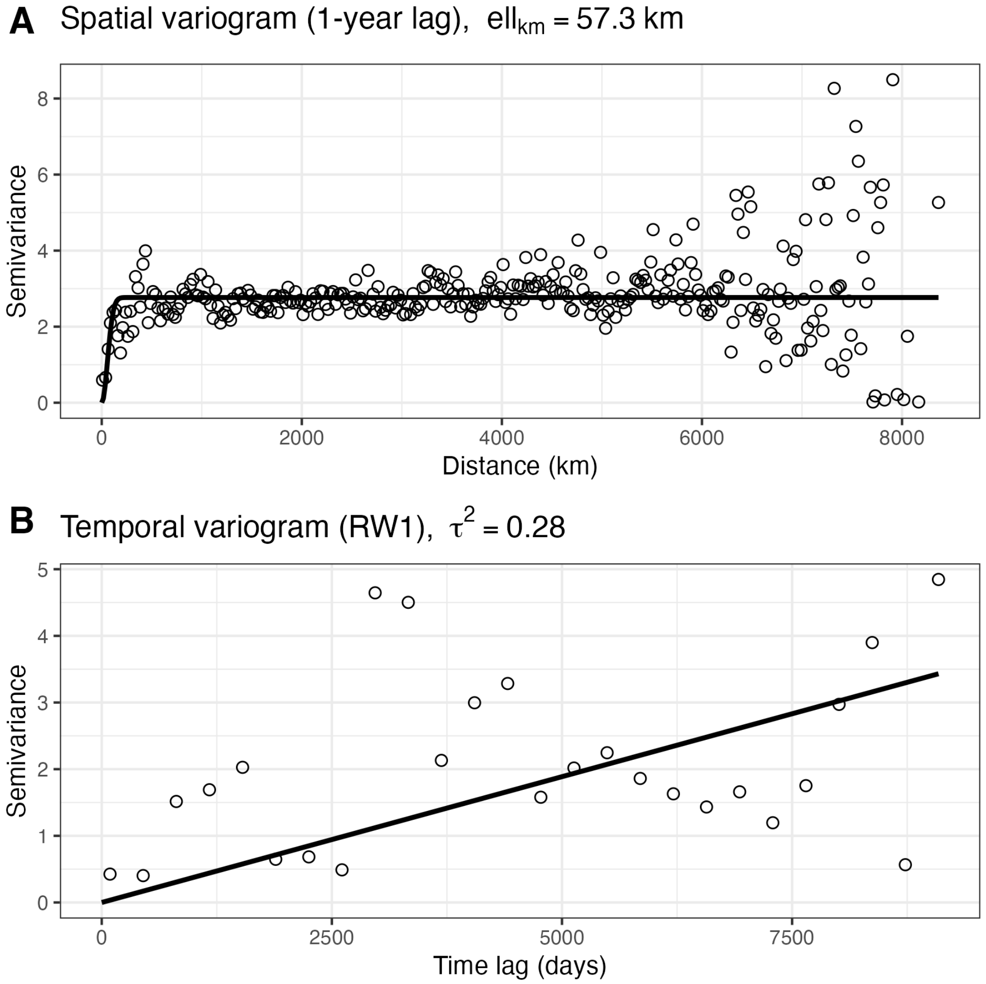

Figure S4: (A) Empirical spatial variogram for all validated and candidate *k13* mutations combined at an approximately one-year temporal lag, with a fitted Gaussian covariance model used to estimate the spatial correlation length scale. (B) Empirical temporal variogram with a fitted first-order random-walk model (intercept fixed at zero) used to estimate temporal variance. Points denote empirical semivariances and solid lines indicate fitted models.

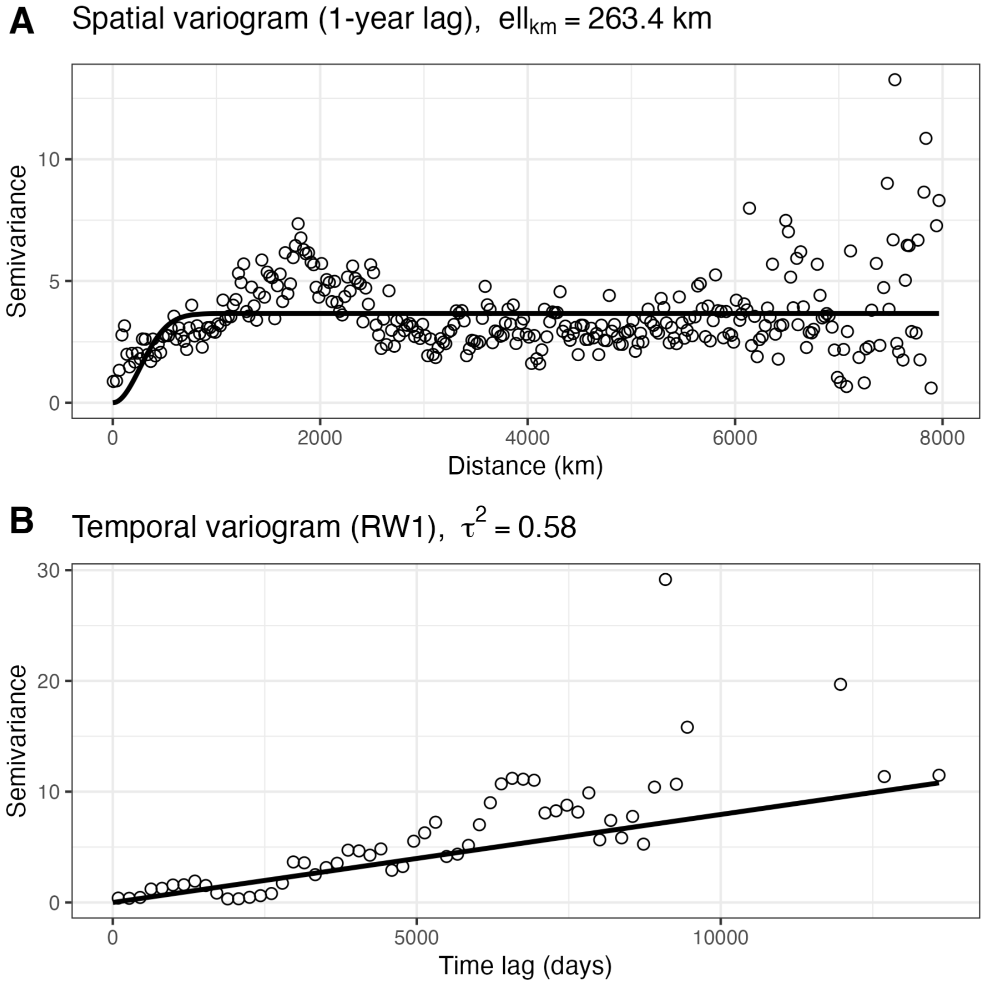

Figure S5: (A) Empirical spatial variogram for *crt* 76T and *mdr1* 86Y combined at an approximately one-year temporal lag, with a fitted Gaussian covariance model used to estimate the spatial correlation length scale. (B) Empirical temporal variogram with a fitted first-order random-walk model (intercept fixed at zero) used to estimate temporal variance. Points denote empirical semivariances and solid lines indicate fitted models.

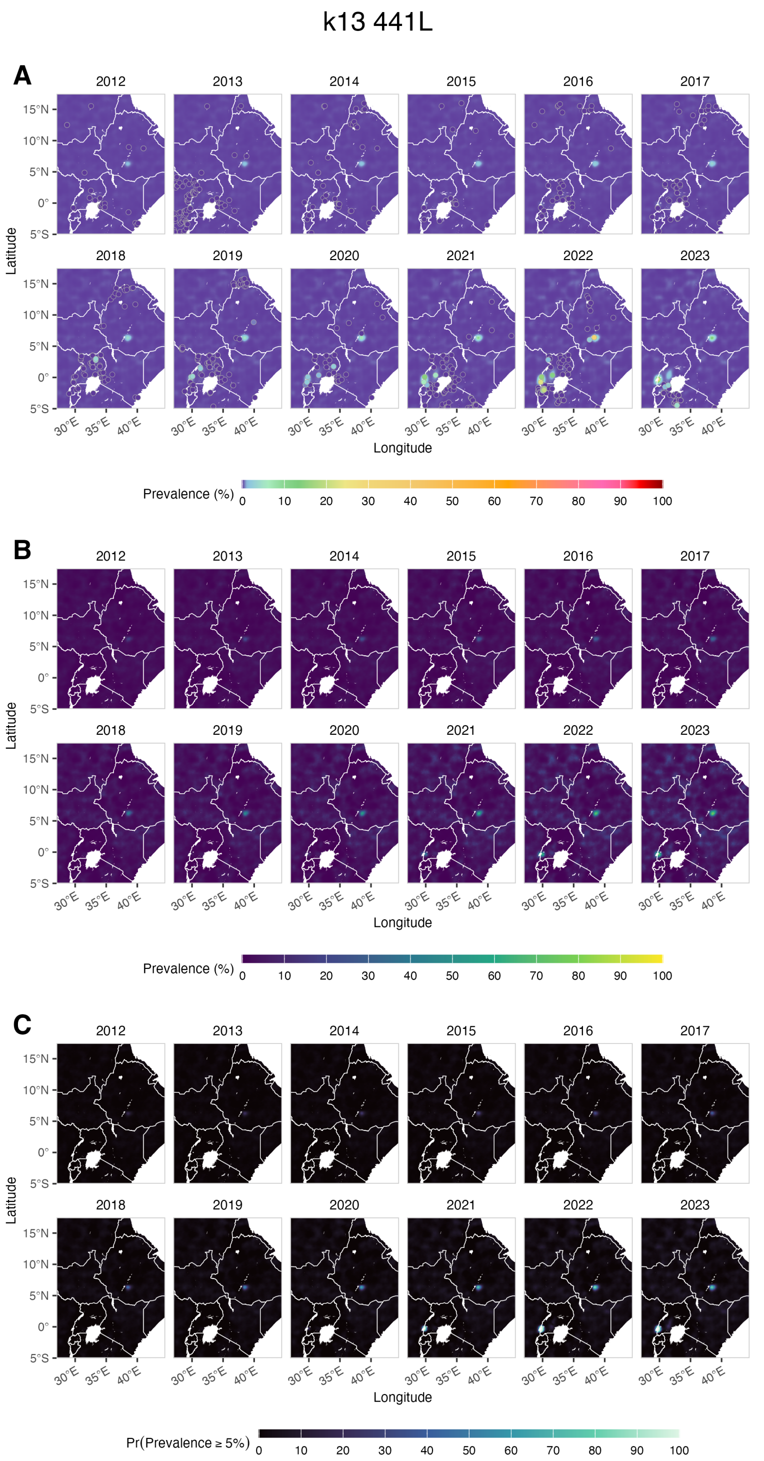

Figure S6: Predicted median prevalence surface with observed survey data overlaid (A), the width of the 95% credible interval (upper minus lower bound) representing predictive uncertainty (B), and the probability that prevalence exceeds 5% (C) for the 441L mutation in East Africa.

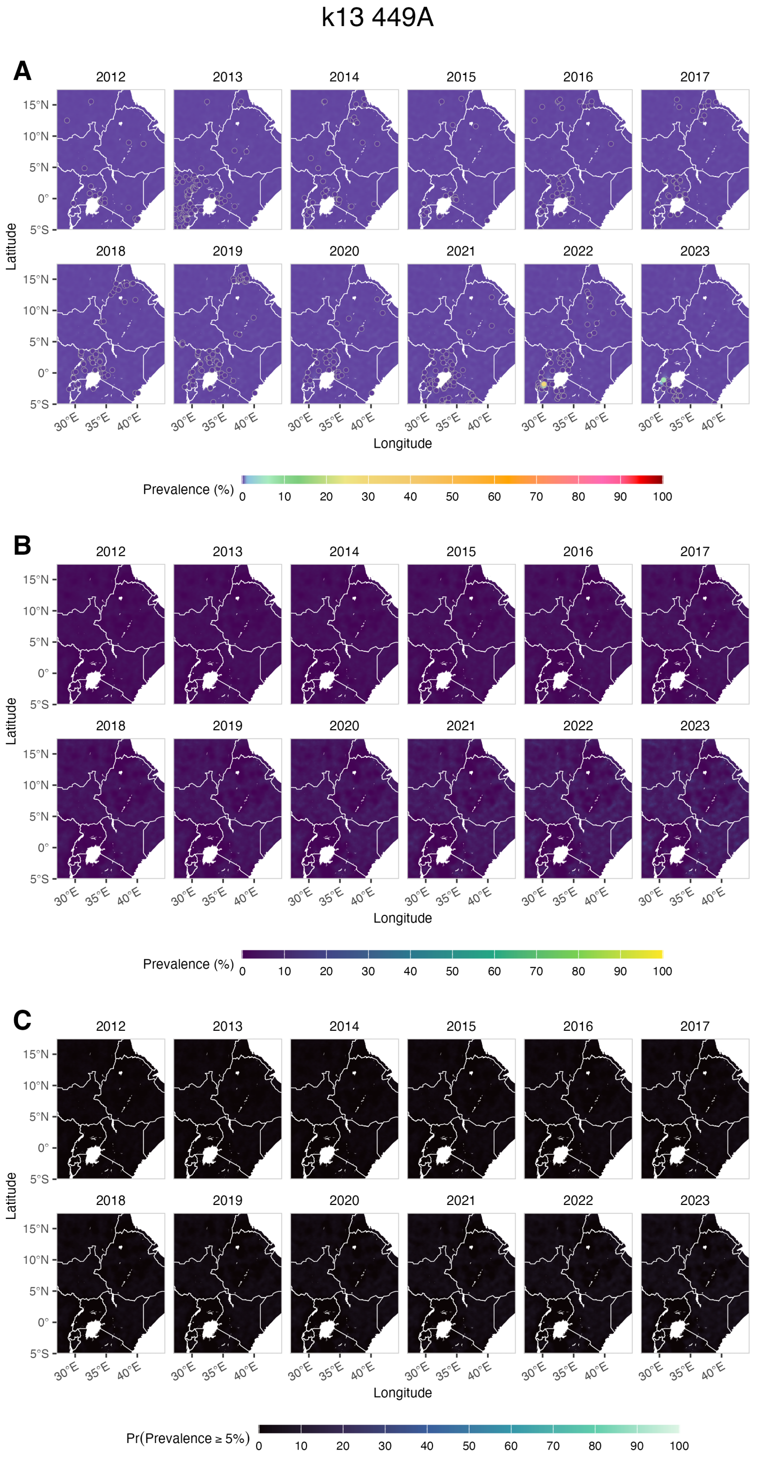

Figure S7: Predicted median prevalence surface with observed survey data overlaid (A), the width of the 95% credible interval (upper minus lower bound) representing predictive uncertainty (B), and the probability that prevalence exceeds 5% (C) for the 449A mutation in East Africa.

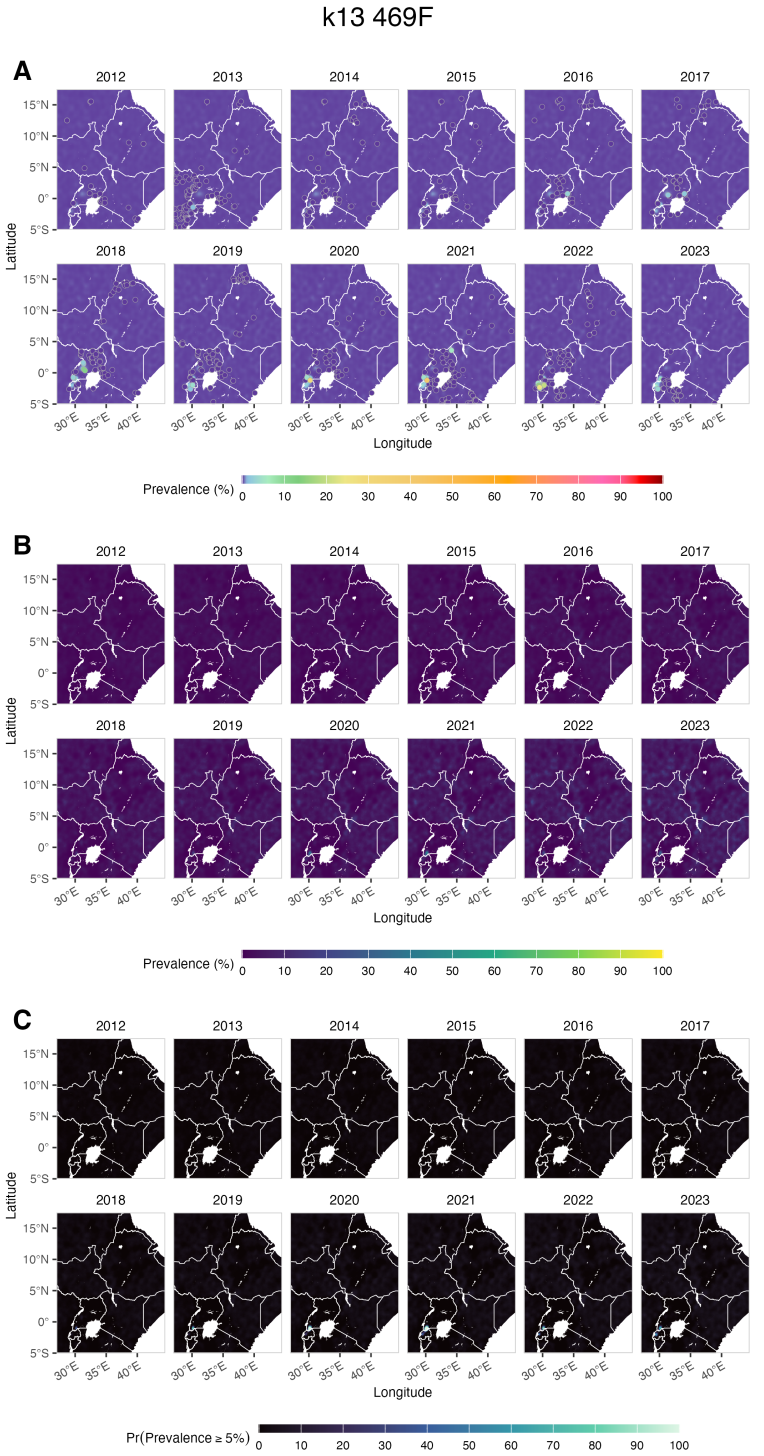

Figure S8: Predicted median prevalence surface with observed survey data overlaid (A), the width of the 95% credible interval (upper minus lower bound) representing predictive uncertainty (B), and the probability that prevalence exceeds 5% (C) for the 469F mutation in East Africa.

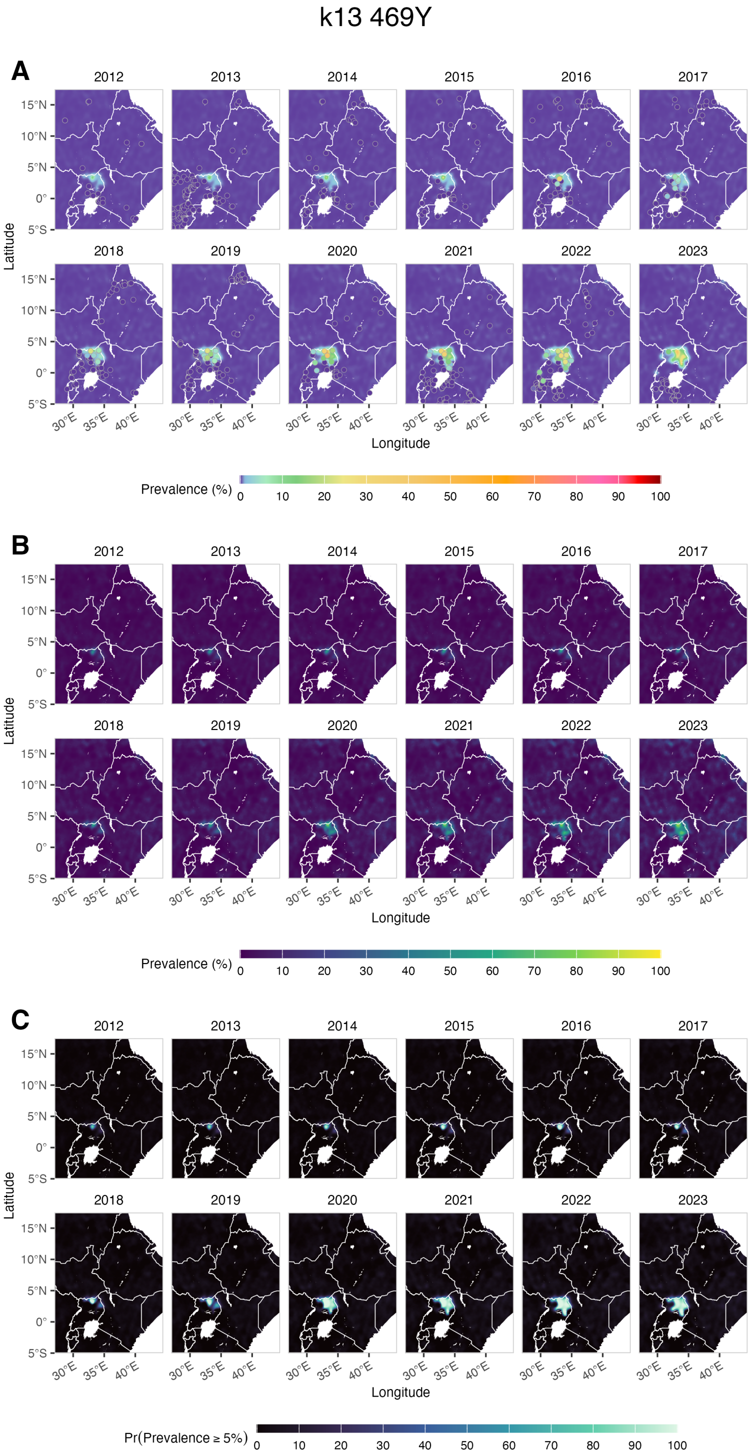

Figure S9: Predicted median prevalence surface with observed survey data overlaid (A), the width of the 95% credible interval (upper minus lower bound) representing predictive uncertainty (B), and the probability that prevalence exceeds 5% (C) for the 469Y mutation in East Africa.

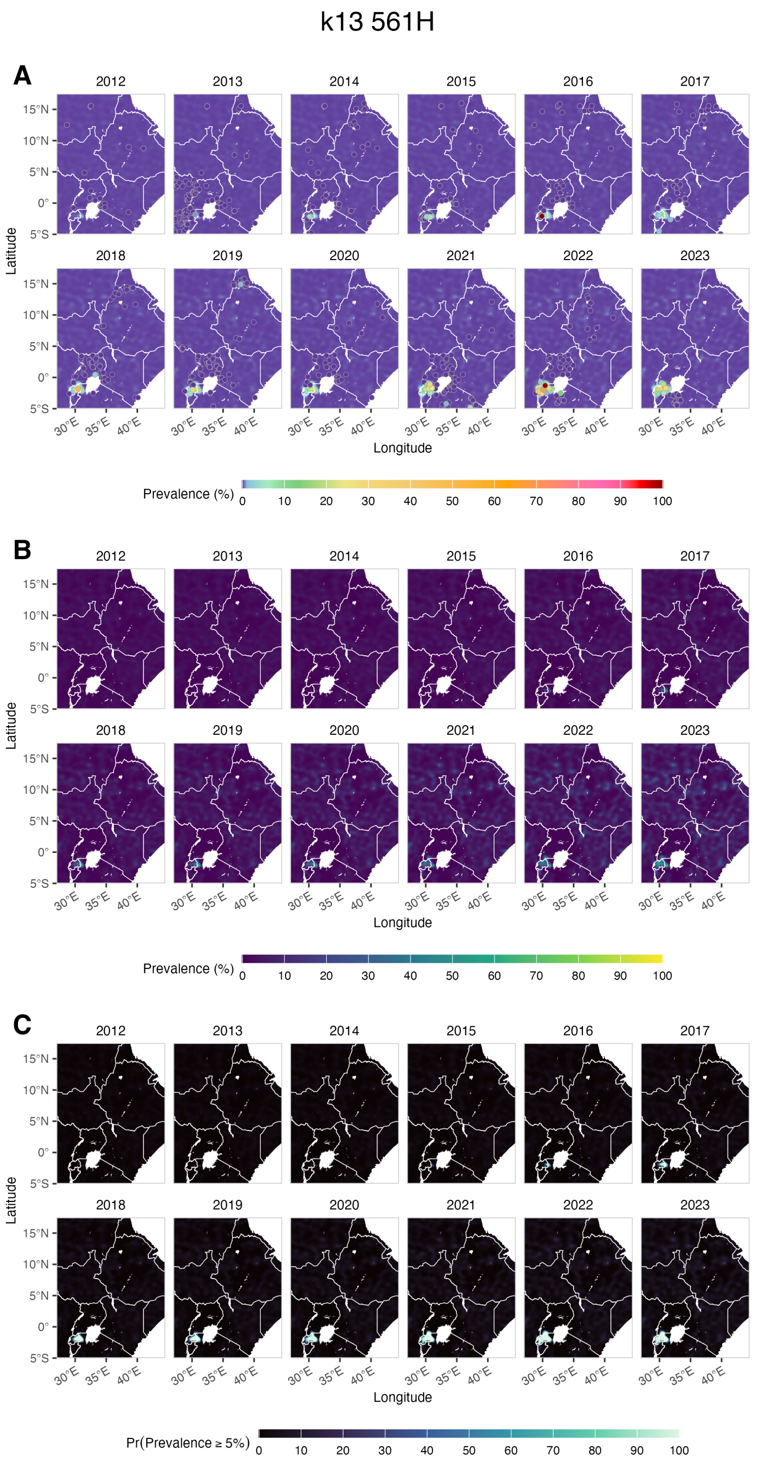

Figure S10: Predicted median prevalence surface with observed survey data overlaid (A), the width of the 95% credible interval (upper minus lower bound) representing predictive uncertainty (B), and the probability that prevalence exceeds 5% (C) for the 561H mutation in East Africa.

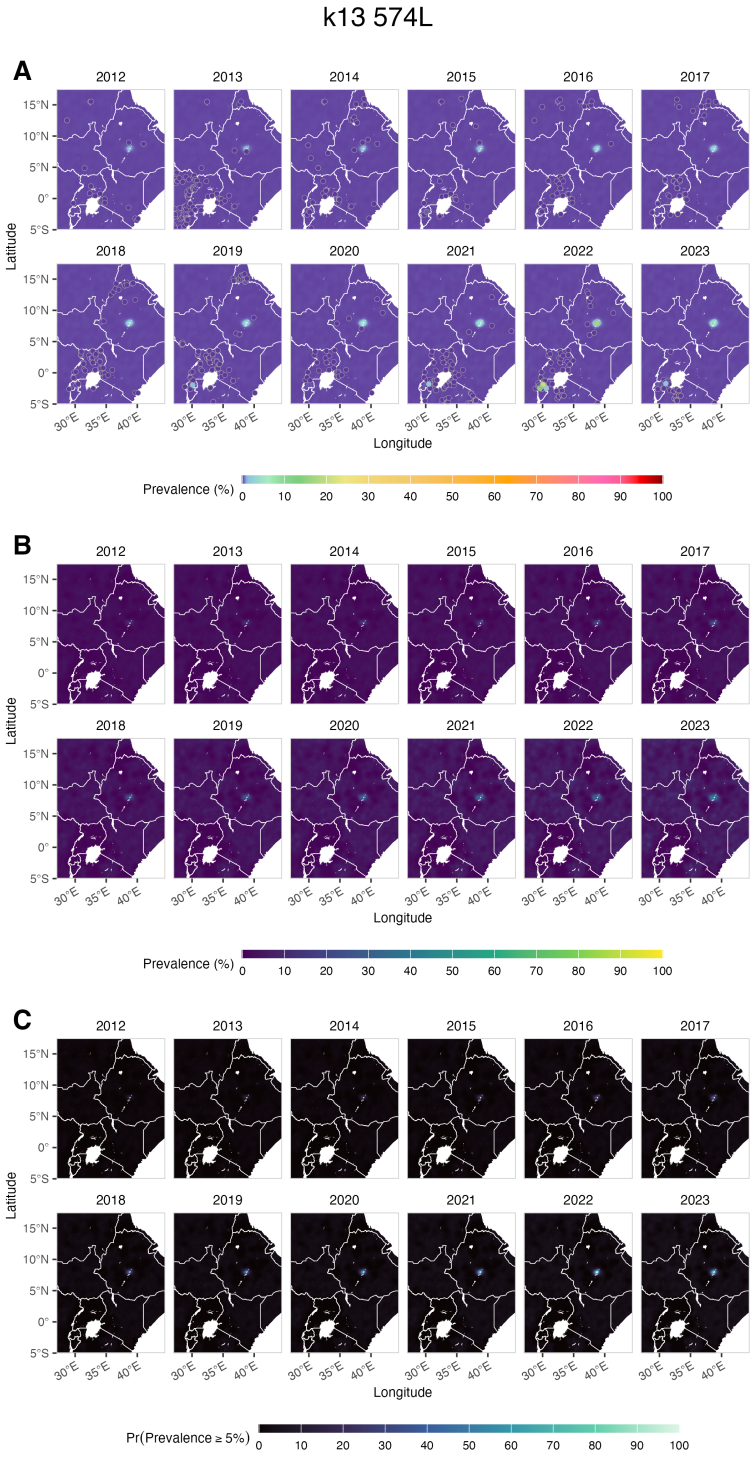

Figure S11: Predicted median prevalence surface with observed survey data overlaid (A), the width of the 95% credible interval (upper minus lower bound) representing predictive uncertainty (B), and the probability that prevalence exceeds 5% (C) for the 574L mutation in East Africa.

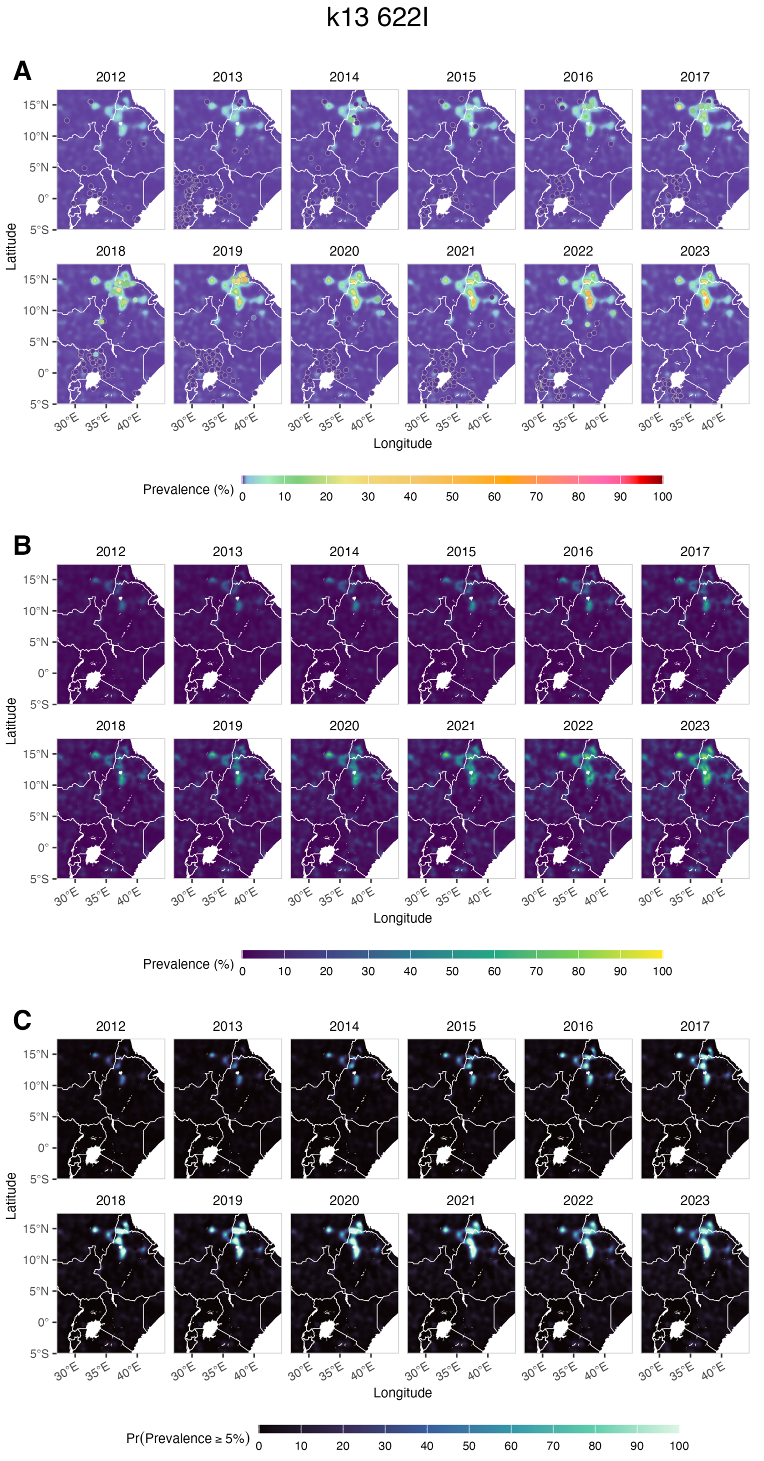

Figure S12: Predicted median prevalence surface with observed survey data overlaid (A), the width of the 95% credible interval (upper minus lower bound) representing predictive uncertainty (B), and the probability that prevalence exceeds 5% (C) for the 622I mutation in East Africa.

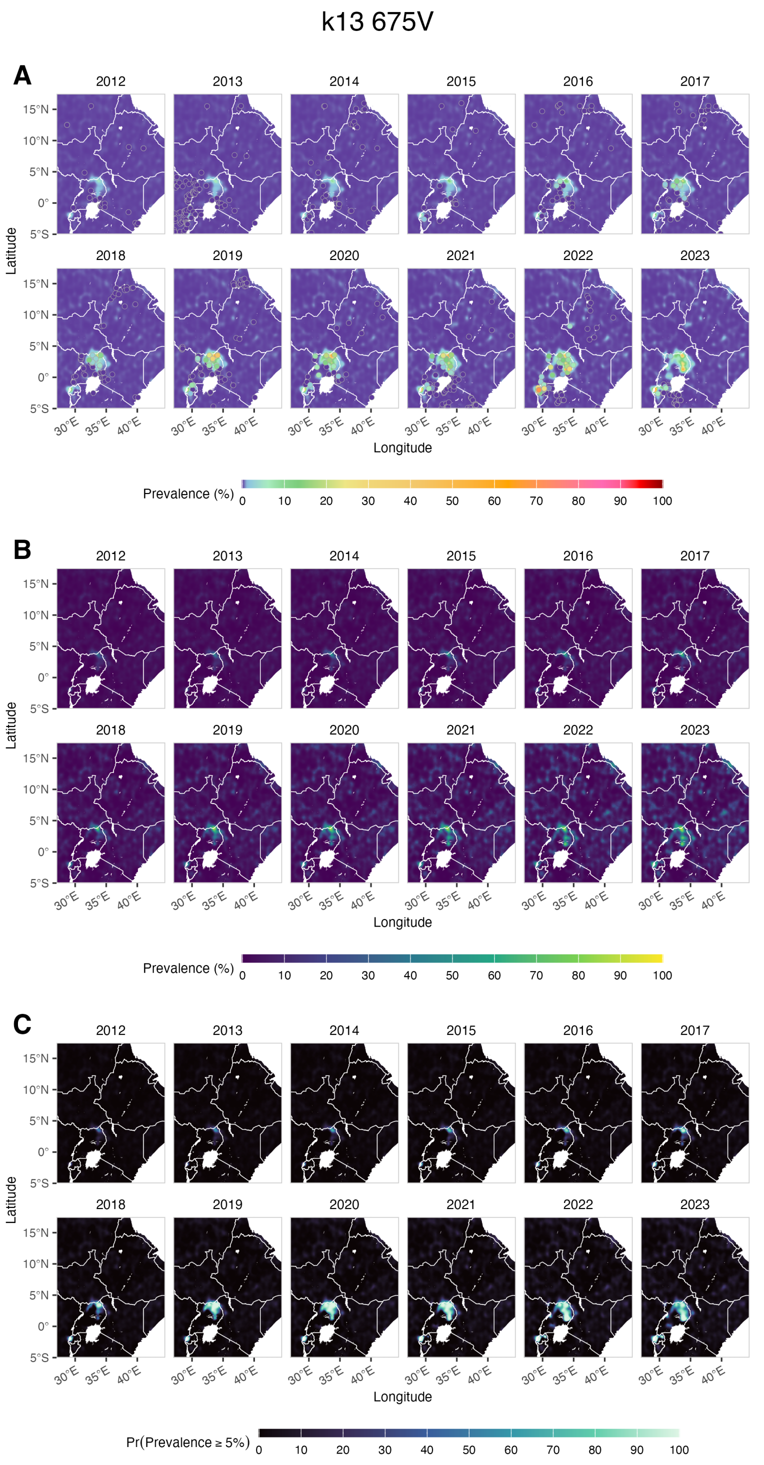

Figure S13: Predicted median prevalence surface with observed survey data overlaid (A), the width of the 95% credible interval (upper minus lower bound) representing predictive uncertainty (B), and the probability that prevalence exceeds 5% (C) for the 675V mutation in East Africa.

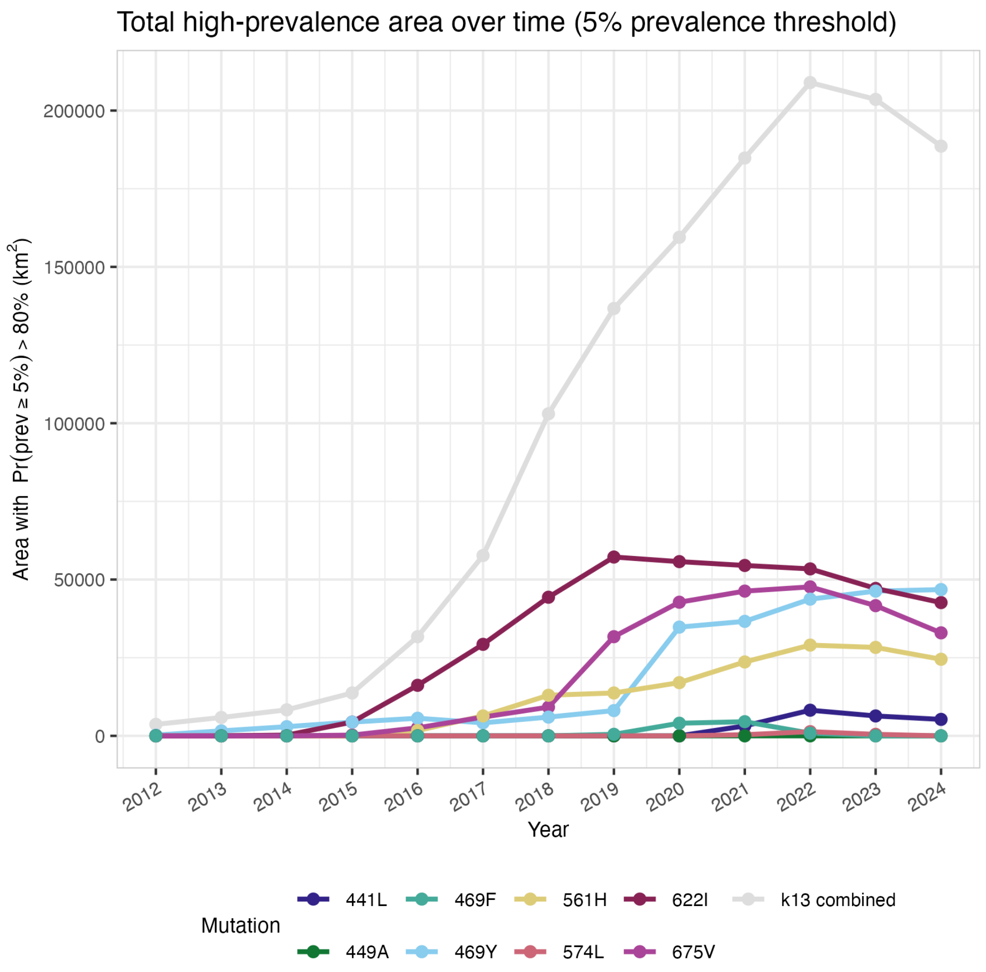

Figure S14: For each year, we count all pixels where the exceedance probability with threshold 5% (Pr(Prevalence > 5%)) is larger than 80%. The sum of those pixels gives a single total-area estimate per year for each mutation with data.

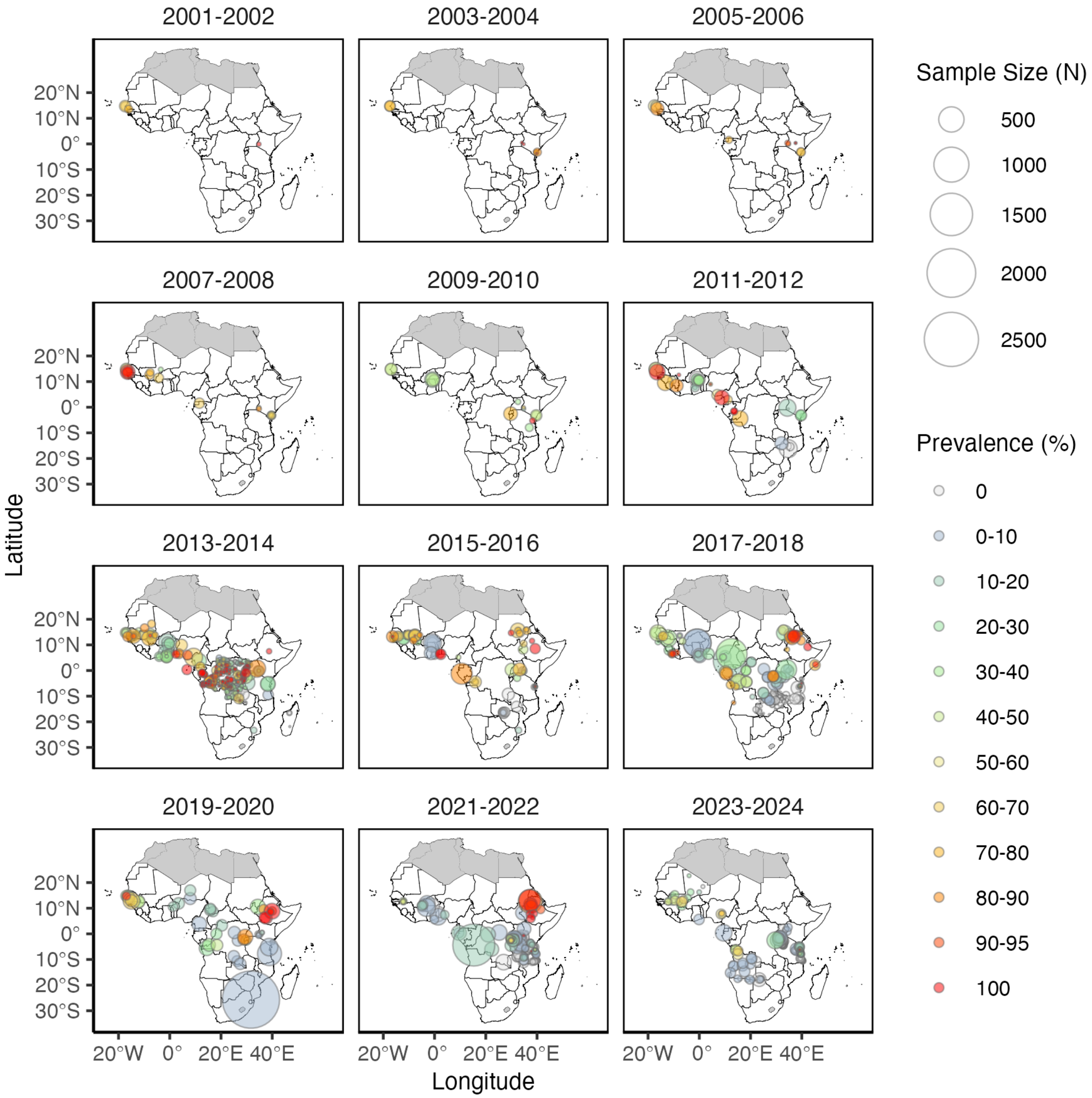

Figure S15: Map of all *crt* 76T datapoints from 2001 to 2024.

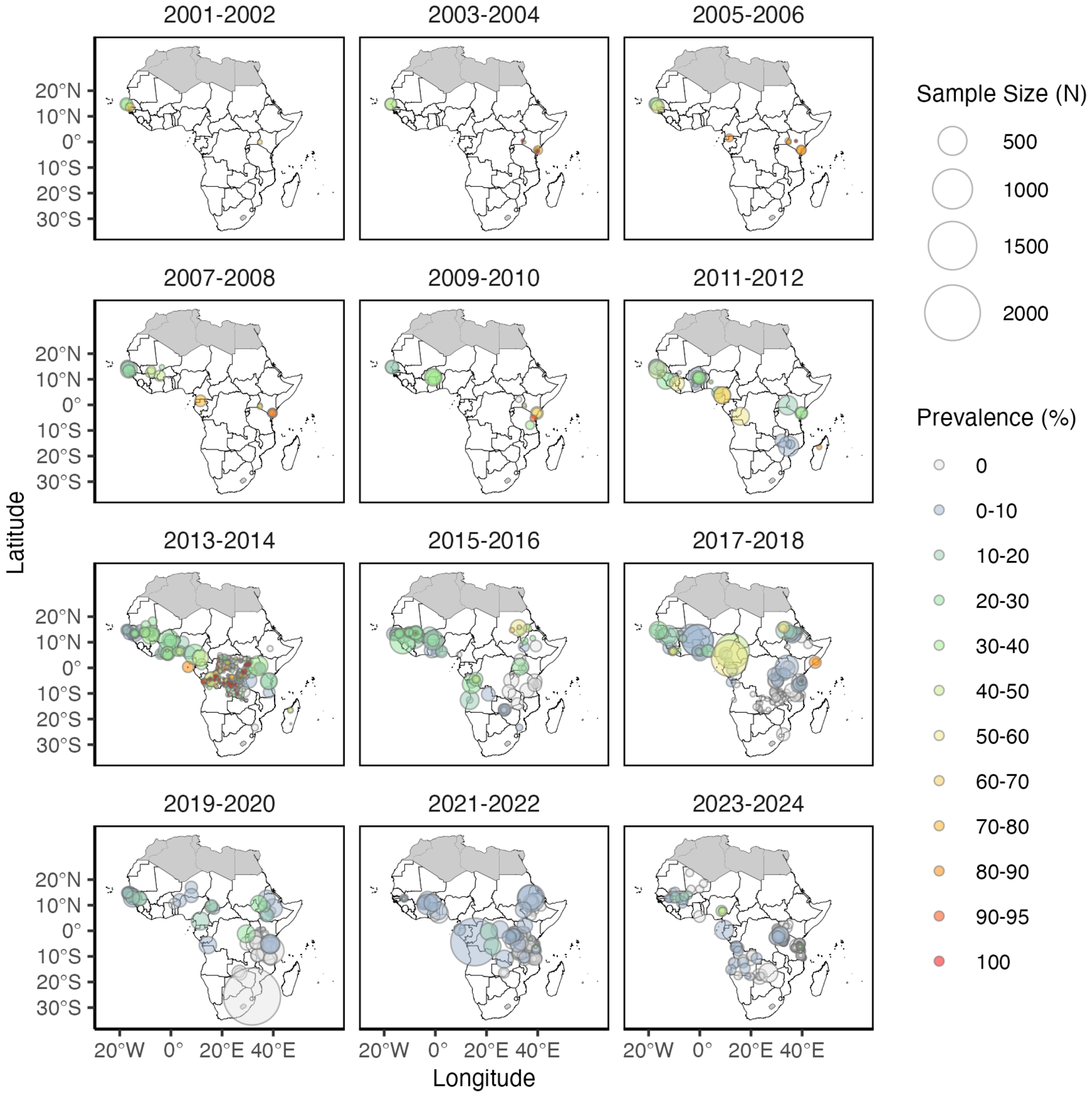

Figure S16: Map of all *mdr1* 86Y datapoints from 2001 to 2024.

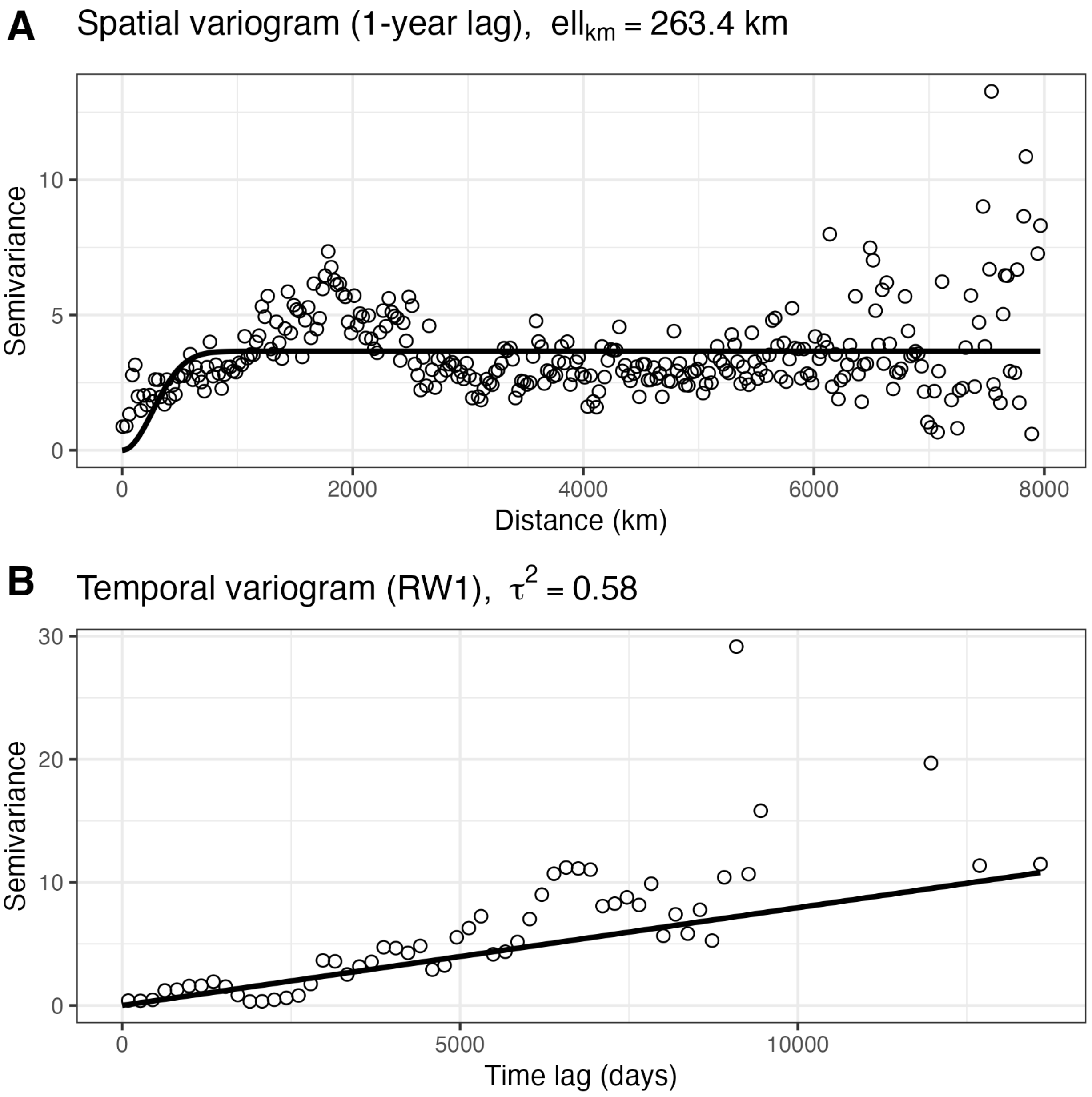

Figure S17: (A) Empirical spatial variogram for *crt* 76T and *mdr1* 86Y mutations combined at an approximately one-year temporal lag, with a fitted Gaussian covariance model used to estimate the spatial correlation length scale. (B) Empirical temporal variogram with a fitted first-order random-walk model (intercept fixed at zero) used to estimate temporal variance. Points denote empirical semivariances and solid lines indicate fitted models.

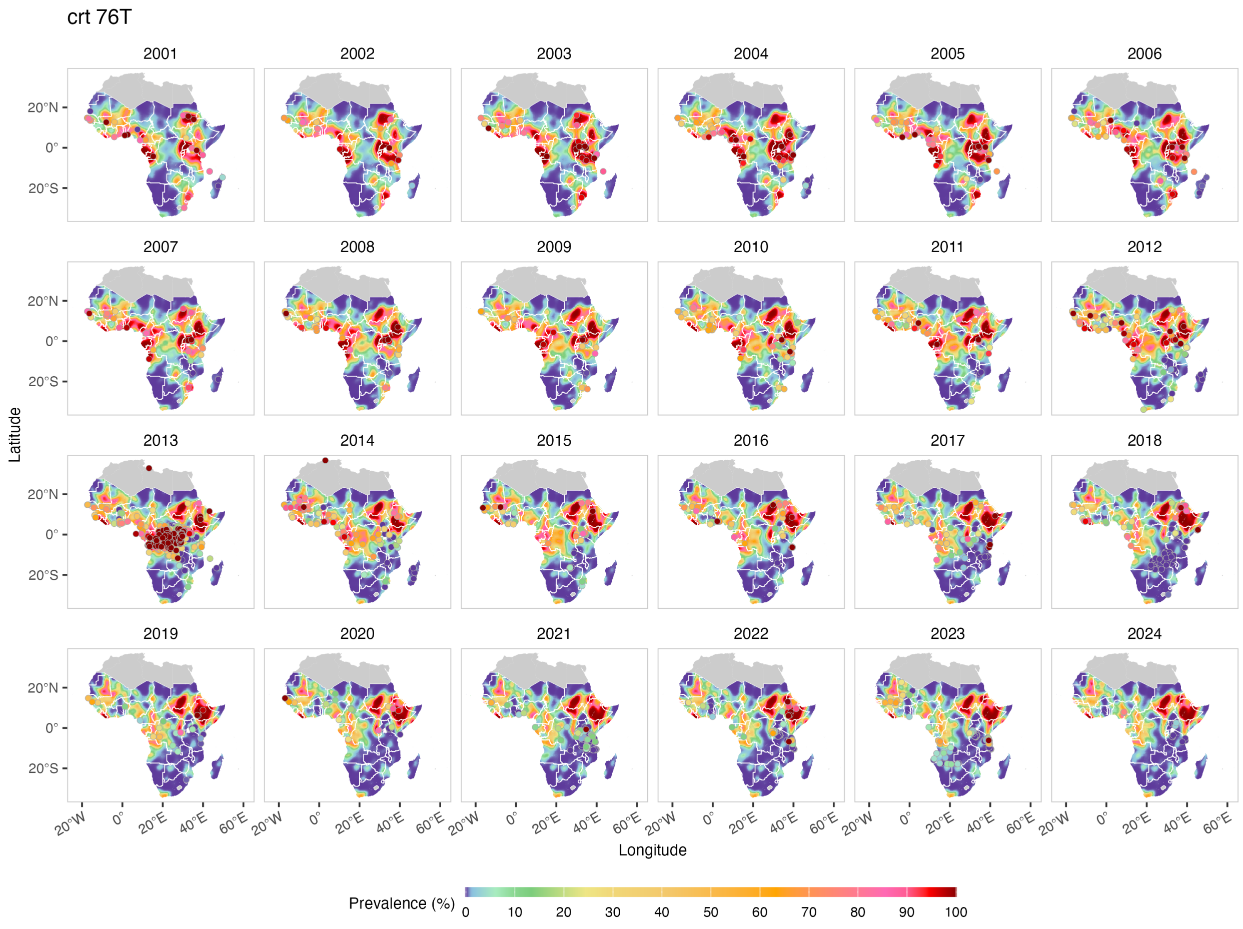

Figure S18: Predicted median prevalence of *crt* 76T across Africa from 2001 to 2024 with datapoints.

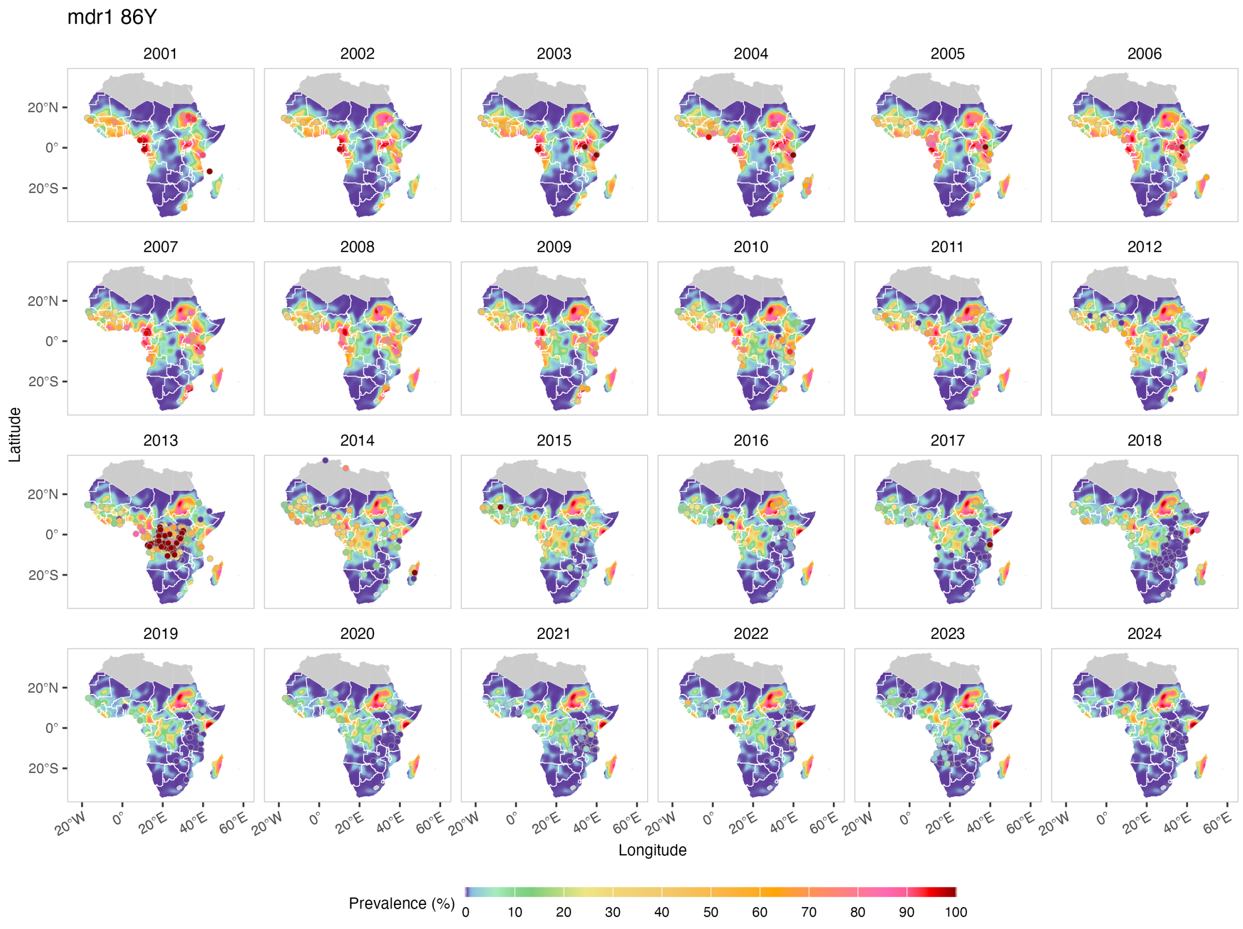

Figure S19: Predicted median prevalence of *mdr1* 86Y across Africa from 2001 to 2024 with datapoints.
